# Biomedical Applications of Silver Nanoparticles: An Up-to-Date Overview

**DOI:** 10.3390/nano8090681

**Published:** 2018-08-31

**Authors:** Alexandra-Cristina Burdușel, Oana Gherasim, Alexandru Mihai Grumezescu, Laurențiu Mogoantă, Anton Ficai, Ecaterina Andronescu

**Affiliations:** 1 Faculty of Engineering in Foreign Languages, University Politehnica of Bucharest, 313 Splaiul Independenței, Bucharest 060042, Romania; alexandra_burdu@yahoo.com.sg; 2 Department of Science and Engineering of Oxide Materials and Nanomaterials, Faculty of Applied Chemistry and Materials Science, University Politehnica of Bucharest, 1-7 Gheorghe Polizu Street, Bucharest 011061, Romania; oana.fufa@gmail.com (O.G.); grumezescu@yahoo.com (A.M.G.); anton.ficai@upb.ro (A.F.); 3 Lasers Department, National Institute for Lasers, Plasma and Radiation Physics, 409 Atomiștilor Street, Magurele 077125, Romania; 4 Research Center for Microscopic Morphology and Immunology, University of Medicine and Pharmacy of Craiova, 2 Petru Rareș Street, Craiova 200349, Romania; laurentiu_mogoanta@yahoo.com

**Keywords:** silver nanoparticles, biomedical applications, biological interactions, biofunctional performances, intrinsic anti-inflammatory activity, antimicrobial efficiency

## Abstract

During the past few years, silver nanoparticles (AgNPs) became one of the most investigated and explored nanotechnology-derived nanostructures, given the fact that nanosilver-based materials proved to have interesting, challenging, and promising characteristics suitable for various biomedical applications. Among modern biomedical potential of AgNPs, tremendous interest is oriented toward the therapeutically enhanced personalized healthcare practice. AgNPs proved to have genuine features and impressive potential for the development of novel antimicrobial agents, drug-delivery formulations, detection and diagnosis platforms, biomaterial and medical device coatings, tissue restoration and regeneration materials, complex healthcare condition strategies, and performance-enhanced therapeutic alternatives. Given the impressive biomedical-related potential applications of AgNPs, impressive efforts were undertaken on understanding the intricate mechanisms of their biological interactions and possible toxic effects. Within this review, we focused on the latest data regarding the biomedical use of AgNP-based nanostructures, including aspects related to their potential toxicity, unique physiochemical properties, and biofunctional behaviors, discussing herein the intrinsic anti-inflammatory, antibacterial, antiviral, and antifungal activities of silver-based nanostructures.

## 1. Introduction

In the past few decades, tremendous interest and substantial research efforts were directed toward the biomedical evaluation and revaluation of metallic nanoparticles derived from noble metals, such as silver and gold, thanks to their specific and genuine chemical, biological, and physical properties [1,2]. In particular, impressive attention was oriented toward the biomedicine-related assessment of silver nanoparticles (AgNPs), which first attracted worldwide attention as unconventional antimicrobial agents [3,4,5]. Even though there is limited information regarding the toxicity and in vivo biological behavior of AgNPs, these nanostructures were used for a long time as antibacterial agents in the health industry [6,7], cosmetics [8,9], food storage [10,11], textile coatings [12,13], and some environmental applications [14,15,16]. AgNPs are a class of zero-dimensional materials with distinctive morphologies, having a size ranging from 1 nm to 100 nm [17].

As to the methods of obtaining AgNPs, different strategies were successfully used, thanks to the intrinsic versatility of silver metal and silver-based compounds, including physical [18,19], chemical [20,21], physicochemical [22,23], and biological synthesis approaches [24,25]. However, given the facile and safe process, reduced economic implications, and repeatability and reproducibility of experimental results, the method most used in the preparation of AgNPs is represented by the chemical reduction of silver salts by sodium citrate or sodium borohydrate [26,27]. In addition to their intrinsic antimicrobial-related applications, AgNPs were thoroughly explored thanks to their beneficial size-related physicochemical effects exhibited in novel electronic, magnetic, catalytic, and optical materials [28,29].

Special interest is oriented toward improving the stability of AgNPs, since a particular limitation of their antimicrobial-related use arises from their instability in bacteria-rich environments, and consequently, diminution or deprivation of their anti-pathogenic activity. In order to improve the stability of AgNPs in solution, many inorganic and organic [30,31], synthetic and natural [32,33], and biotic and abiotic materials were used as capping agents [34].

Though the precise anti-pathogenic mechanism of silver nanoparticles remains to be clarified, it is postulated that nanosilver-based systems exert their antimicrobial effects through the following phenomena: (a) microbial membrane damage, caused by the physicochemically guided attachment of AgNPs on the cell surface, and subsequent structural and functional alterations (such as gap formation, membrane destabilization, membrane piercing, and cytoplasm leakage); and (b) microbial sub-cellular structure damage, caused by the release of free Ag^+^ ions and subsequent reactive oxygen species (ROS) generation or essential macromolecule (proteins, enzymes, and nucleotides) inactivation [35,36,37]. Still, the most remarkable mechanistic mode of AgNP-based antimicrobial effects is represented by their adhesion to microbial cells, ROS and free-radical generation, microbial wall piercing and penetration inside cells, and modulation and modification of microbial signal-transduction pathways [38]. Metallic silver ions are strong antimicrobials themselves, but they are easily isolated by phosphate and chloride functions, proteins, and different cellular components [39]. The intrinsic biocide or biostatic activity of AgNPs is strongly influenced by different physicochemical features, including morphology, size, oxidation and dissolution states, surface charge, and surface coating [37,40].

The effectiveness of nanosilver-based biomaterials as promising antimicrobial agents was experimentally assessed against a wide range of medically relevant planktonic and sessile pathogenic microorganisms, including bacteria [41,42], viruses [43,44], fungi, and yeasts [45,46]. The impressive antimicrobial activity of AgNPs is a solid starting point for the design, development, and implementation of new and performance-enhanced nanosilver-based biomedical products, such as anticancer agents, drug-delivery platforms, orthopedic materials and devices [47], bandages, antiseptic sprays, and catheters [48]. As a consequence of the impressive applicability of AgNPs in the fields of nanotechnology, biomedicine, and environment, there is a continuous need for the development of cost-effective methods for the synthesis of AgNPs [49]. The translation of silver-based nanotechnology to clinical applications requires not only the development of safe, simple, eco-friendly, and cost-effective methods for the synthesis of silver nanoparticles, but also a thorough understanding of the related physicochemical particularities, in vitro and in vivo effects, biodistribution, safety control mechanisms, pharmacokinetics, and pharmacodynamics of AgNPs [48].

## 2. Antibacterial Characteristics of Silver Nanoparticles

Silver nanoparticles attracted tremendous interest in the biomedical field, thanks to their attractive and unique nano-related properties, including their high intrinsic antimicrobial efficiency and non-toxic nature. Among the manifold potential applications of AgNPs in this particular domain, impressive attention and efforts were lately directed toward their promising implications in wound dressing, tissue scaffold, and protective clothing applications [50,51]. Some essential aspects related to the specific antimicrobial characteristics of AgNPs implies their intrinsic physical and chemical properties, which include maintaining the nanoscale size of AgNPs, improving their dispersion and stability, and avoiding aggregation [52]. There are many studies which experimentally proved that the anti-pathogenic activity of AgNPs is better than that exhibited by silver ions [53].

**Figure 1:**
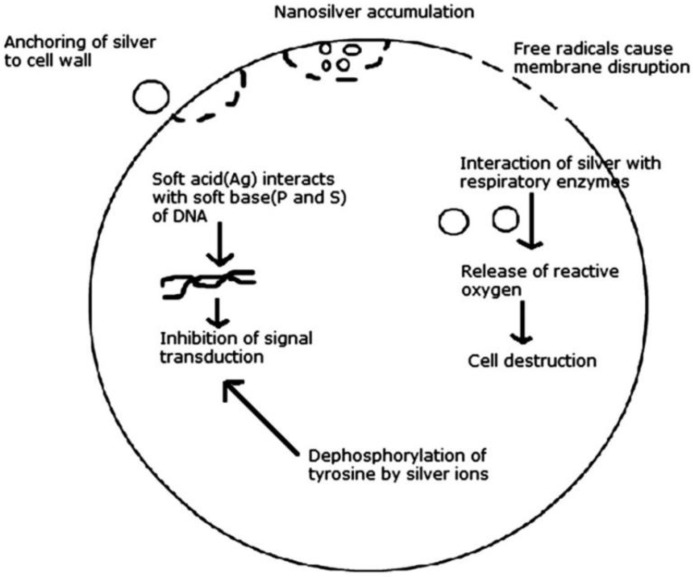
Various modes of action of silver nanoparticles (AgNPs) on bacteria [57].

A major concern of the worldwide healthcare system is represented by the alarming and emerging phenomenon of pathogenic drug-resistant occurrence. Therefore, AgNPs represent potent candidates for the nanotechnology-derived development of novel and effective biocompatible nanostructured materials for unconventional antimicrobial applications [54]. Thanks to their intrinsic broad bactericidal effects exhibited against both Gram-negative and Gram-positive bacteria and their physicochemical properties, AgNPs are one of the most used metallic nanoparticles in modern antimicrobial applications [55]. Different studies reported that AgNPs interact with the bacterial membrane and penetrate the cell, thus producing a drastic disturbance regarding proper cell function, structural damage, and cell death [56]. We included in Figure 1 distinctive mechanisms described during the interaction of AgNPs with bacterial cells [57].

Currently, it is shown that AgNPs can be successfully used to design and develop improved wound and burn dressings, thanks to the intrinsic antibacterial and anti-inflammatory effects of sole metallic nanoparticles [58]. Given the mutation-resistant antimicrobial activity related to nanosilver-based biomaterials, AgNPs are used in various pharmaceutical formulations as burn ointments, antibacterial clothing, and coatings for medical devices [59]. Different studies proved that the stabilization of AgNPs against dissolution and/or agglomeration can be achieved by using various capping agents, such as sodium citrate, polyvinylpyrrolidone (PVP), or polyethylene glycol (PEG) [60]. The stability of AgNPs was previously investigated, where the reported data indicated that their stability actively influences their toxicity [60].

The most important physicochemical parameters that affect the antimicrobial effects exhibited by AgNPs include size [61], shape [62], concentration [63], surface charge [64], and colloidal state [65]. As mentioned before, AgNPs display their intrinsic enhanced antimicrobial activity through various mechanisms [66].

It is also worth mentioning that the AgNP-based treatment of human cell cultures may induce cytotoxicity [67], inflammatory responses in a cell-type-dependent manner, and genotoxicity [66]. Thanks to their intrinsic capacity to provide stimuli-dependent responses through the specific modification of their optical properties, chemical environments, high molar absorptivity [68], and the many sorption sites found on their extensive surface [69], AgNPs are also used in various analytical applications. There are manifold research studies which particularly describe the biocide activity of silver itself [70].

The current state of the art relies on the beneficial conjunction between antimicrobial silver nanoparticles and natural or synthetic polymers, in the modern attempt to diminish or even eliminate the microbial contamination and colonization processes [71].

The major advantage of nanosilver-based biomaterials designed for unconventional antibacterial applications is related to their intrinsic anti-pathogenic effects exhibited against both planktonic and biofilm-organized microorganisms. The bactericidal activity of AgNPs is attributed to silver cations, which possess the ability to specifically bind to thiol groups of bacterial proteins, disrupting their physiological activity and leading to cell death. The effects of AgNPs on bacterial DNA were not analyzed in detail with respect to possible DNA lesions and antibacterial action that occur after AgNPs treatment. Silver nanoparticles exert their bactericidal activity through a Trojan-horse mechanism, since their initial binding to the cell surface leads to permeability alteration and cellular respiration impairment, followed by cell-barrier penetration and intracellular metallic silver ion release.

In order to successfully apply nanosilver-based systems as effective antibacterial agents, it is important to thoroughly understand their action against bacterial cells and bacterial biofilms [72]. In addition to their improved efficiency against planktonic bacteria (as discussed above), AgNPs also possess bactericide or bacteriostatic activity against biofilm-organized microorganisms. The antibacterial effects exhibited by silver-based nanosystems against biofilm-organized bacteria may be due to intrinsic activity against isolated or block cells, destabilization or disruption of the exopolymeric substances within the extracellular biofilm matrix, or interfering with bacterial signaling molecules [73,74,75]. There are ongoing discussions regarding the role of Ag ions released from AgNPs and their related toxic effect on microorganisms. Many researchers stated that the toxicity of AgNPs is due to the nanoparticles themselves, whereas others provided evidence that silver ions released from AgNPs play a crucial role during antimicrobial activity. Following the release of silver ions from AgNPs, antibacterial activity is initiated by metallic cations, rather than by metallic nanoparticles [76].

## 3. Silver Nanoparticles for Drug-Delivery Systems

In medicine, the pharmacokinetics and pharmacodynamics of drugs are as important as their intrinsic therapeutic effects [77]. Since the specific and selective delivery and action of therapeutic agents became one of the most studied topics for improving current human healthcare practice, nanoparticles received tremendous attention regarding the design and development of novel and enhanced drug-delivery systems [78]. In particular, AgNP-based nanosystems were evaluated as suitable carriers of various therapeutic molecules, including anti-inflammatory [79,80], anti-oxidant [81,82], antimicrobial [83,84], and anticancer [32,85] biosubstances.

In order to provide a specific therapeutic effect in human or animal organisms, it is essential to consider the process or method applied during the administration of the selected pharmaceutical compound [86]. For obtaining novel and performance-enhanced drug-delivery systems responsive to thermal, optical, or pH modulations to target inflammatory, infectious, and malignant ailments, hybrid molecular units consisting of AgNPs were successfully chosen, especially thanks to their exceptional biocompatibility and viable features for nanoscale-derived therapeutic settings [87].

As a consequence of the difficulty encountered during AgNP synthesis and the concerns regarding the toxicity and reduced stability of nanosilver-based systems when functionalized according to conventional salt-aging techniques, silver is not extensively used in nanoparticle-based drug-delivery applications, instead being replaced with gold or other nanomaterials [88]. An excellent triggerable and tunable nanosystem for drug-delivery applications should be easy to develop from readily available components, exhibit optimal responsiveness, and be compatible with more than one trigger [89]. Moreover, such a particular drug-delivery [90] platform should not only provide suitable and adjustable drug loading and releasing profiles, but should also enable [70] maximal therapeutic efficiency at concentrations below that of the sole biosubstance with side-effect minimization [91,92].

Thanks to their intrinsic anticancer activity [93], AgNPs attracted special attention for this particular domain, and were successfully evaluated as effective anti-tumor drug-delivery systems [94], acting either as passive [95,96] or active [97,98] nanocarriers for anticancer drugs. For the preparation of biocompatible AgNPs, different strategies were used, such as organic-water two-phase synthesis [99,100,101], micro-emulsion [102,103,104], radiolysis [105,106], and most commonly, reduction in aqueous solution [94,107,108]. Impressive attention, scientific knowledge, and financial support were lately oriented toward the formulation of AgNP-based drug-delivery platforms, thanks to the intrinsic features of nanosilver, including its capacity to bind a wide range of organic molecules, its tunable and strong absorption properties, and its low toxicity [109]. Recent studies evidenced the potential use of AgNPs as vaccine and drug carriers for specific and selective cell or tissue targeting [109]. In addition to the great optical properties of AgNPs (governed by specific surface plasmon resonance and localized surface plasmon resonance) [110,111,112], the recent improvements in AgNP biocompatibility and stability via surface modification strongly recommend nanostructured systems based on silver as specific, selective, and versatile candidates for drug-delivery applications [113].

## 4. Silver Nanoparticles for Catheter Modification

Central venous catheters (CVC) were firstly described by Niederhuber in 1982; since then, these devices became important therapeutic tools for diverse clinical conditions requiring malnutrition and replacement therapy (e.g., renal disease and cancer) [114]. CVCs are normally used to provide access for intravenous fluid administration, hemodynamics monitoring, drug-delivery pathways [115], and nutritional support in critically ill patients. Still, these medical devices are also a considerable source of hospital-acquired infections [116], and are considered a specific high-risk category of devices susceptible to microbial contamination and colonization phenomena [117]. A recent study showed that various *Staphhylococcus aureus* strains are responsible for catheter-related infections, and 82% of them are methicillin-resistant strains possessing many genes expressed in biofilm development and bacterial dispersion processes [118].

In order to induce antibacterial effects to clinically relevant materials and devices, AgNPs were extensively explored for the modification of one-dimensional and two-dimensional surfaces [119], such as cotton fabrics [120,121], natural and artificial fibers [122,123,124], thin polymer films [125,126], and wound pads [127,128].

Even if silver (a half-noble metal) is susceptible to quick oxidation processes, the impressive surface-to-volume atomic ratio related to AgNPs accounts for the sustained local supply of Ag^+^ ions at the coating/tissue interface [129]. In recent studies, the role of AgNP-modified catheters as non-toxic devices capable of sustained release of bactericidal silver, exhibiting preventive effects against infection-related complications, was presented [116,130,131]. Given the fact that one of the major groups of organisms that causes device-related infections is represented by coagulase-negative staphylococci (CoNS), the effects exhibited by AgNPs and AgNP-coated catheters against these organisms were intimately studied [38]. Significant inhibitory effects against both Gram-positive and Gram-negative bacterial biofilm development were exhibited by CVCs coated with AgNPs [115,132,133,134].

Because the binding capacity of silver nanoparticles to bacterial cells is influenced by the surface area available for interaction, the bactericidal effects are expected to be size-dependent [135]. Catheters treated with silver ions represent a feasible strategy for reducing dialysis-related infections in patients undergoing peritoneal catheters; however, the antimicrobial efficiency and obtaining methods of Ag^+^ are different [136]. Silver/copper-coated catheters were assessed as a promising solution for preventing methicillin-resistant *Staphylococcus aureus* (MRSA) infections, since their antibacterial activity might be improved by limiting non-specific plasma protein adsorption [137].

The main complication related to urinary catheterization is represented by the occurrence of catheter-associated urinary tract infections (CAUTIs) [138]. It was shown that a polymer matrix impregnated with AgNPs displayed hydrophilic surface properties, resulting in the prevention of bacterial biofilm formation and the deposition of proteins and electrolytes responsible for incrustation and adherence of microorganisms onto the surface [139]. With regards to silicon urethral catheters, Kocuran-capped silver glyconanoparticles were successfully evaluated as effective antibiofilm and antimicrobial coatings [118]. Despite the concerns regarding CVC-related complacency with respect to septic techniques, catheters with antimicrobial properties were taken into consideration as a feasible means of supplying additional protection against microbial contamination, further reducing colonization and infection risks [117].

## 5. Silver Nanoparticles for Dental Applications

Dental caries represent one of the most extensive oral-cavity-related affections worldwide, being also an economic burden [140]. By enhancing the remineralization process and controlling biofilm development, nanotechnology-derived dental-related strategies aim to limit or even eliminate the clinical impact of caries [140]. In addition to their intrinsic highly biocompatible behavior, the materials for dental barrier membranes (DBM), which are often used for efficient alveolar bone reconstruction, must accomplish some specific and additional features and functions [141]. Different metal-coated implants were evaluated against various pathogens responsible for dental-related biofilm formation and subsequent implant failure [142].

In order to prevent the pathogenic contamination of dental implants, proper tooth-brushing techniques, prophylactic antibiotics, and antimicrobial mouthwashes are specifically recommended [143]. A major goal in dentistry is to provide the proper protection of the oral cavity, which represents a pathogenic-susceptible gateway for the entire body [144]. Biofilms developed on dental implant surfaces may additionally cause inflammatory lesions on the peri-implant mucosa, thus increasing the risk of implant failure [145].

Silver was used for centuries in oral care and gained worldwide attention in the 19th century, being a major component in dental amalgams used for tooth restoration [146]. AgNPs were also used in various fields of dentistry, such as dental prostheses, restorative and endodontic dentistry, and implantology [147]. Thanks to their unique properties feasible for different domains of real interest in modern society, silver nanoparticles hold a prominent place in nanomaterial-related restorative, regenerative, and multifunctional biomedicine [148,149].

An attractive strategy embraced by worldwide practitioners in order to provide additional bactericidal effects to general-use dental materials is to modify or embed them with silver-based nanostructures [150]. Though silver has favorable effects in caries prophylaxis in the form of nanosilver diamine fluoride (SDF), the use of this particular compound has some disadvantages, one of the most noticeable effects being represented by tooth staining [151]. By reducing the size of AgNPs, the contact surface will be considerably increased; in this way, the antimicrobial effects of silver would be improved, and the use of nanosilver could prevent black staining in teeth, which usually occurs after the application of SDF [152].

Antibacterial resins could be used in clinical dental applications, both in orthodontics and restorative dentistry [153]. In orthodontics, these resins could be used as bracket or branked bonding materials, while, in restorative dentistry, they could be used as filling or denture base material [153]. Therefore, in order to improve their physico-mechanical properties and antimicrobial effects, a method for incorporating AgNPs into acrylic resin denture-base materials was developed [154].

Because the oral cavity is an active ecosystem usually colonized by various pathogenic microorganisms, dental materials and implants have an increased risk of contamination and subsequent colonization processes [155]. In terms of superior antimicrobial activity, promising results were reported with respect to the incorporation of silver-based nanosystems within adhesive resins [156,157], orthodontic cements [158,159], and dental composites [160,161,162]. In addition to being used as antimicrobial filling agents within multifunctional biomaterials, another attractive and challenging dental application of AgNPs relies on their potential use as biostatic or biocide coatings for conventional titanium-based dental implants [163,164]. Though AgNPs proved to be efficient and effective agents in dental practice, they remain controversial candidates for this specific area of research, due to their variable toxicity in biological systems. Therefore, any potential application of AgNPs in dentistry must include thorough studies regarding the optimal compromise between physicochemical features and biofunctional performance [165].

## 6. Silver Nanoparticles for Wound Healing

Wound infections represent an important clinical challenge, with major impact on patient morbidity and mortality and notable economic implications [166]. Preventing wound dehiscence and surgical-site infection are challenging and essential aspects in current clinical practice [167]. The skin is the most extensive and one of the most complex organs in the human body, but it can be easily affected by different harmful external factors [168]. Physically or chemically induced cutaneous wounds may significantly disturb skin structural and functional integrity at different stages, leading to permanent disability or even death, depending on the severity of the injury [169]. In the past few years, wound infections caused by opportunistic pathogenic microorganism became an important issue during current healthcare practice [170]. The ultimate tendency and ideal desideratum for infected-wound management is represented by fast tissue-recovery processes, accompanied by maximal functionality restoration and minimal scar-tissue formation [171]. The wound-healing process, as any complex pathophysiological mechanism, includes different stages, such as coagulation, inflammation, cellular proliferation, and matrix and tissue remodeling [171].

Since ancient times, silver-based compounds and materials were used for the unconventional and effective control of distinctive infections [172]. Given its intrinsic physicochemical features and biological peculiarities, nanosilver provides a wide range of efficient biocide activities against an impressive diversity of anaerobic and aerobic, Gram-negative and Gram-positive bacterial strains. It is well known that bacterial and mammalian cells poorly absorb metallic or elemental silver, due to its chemical inactivation. Therefore, in order to provide specific antibacterial effects under physiological conditions (including the presence of body fluids or secretions), the ionization of silver is required. After their penetration inside cells, silver ions merge with enzymatic and structural proteins [173]. AgNPs or silver ions used in absorbent wound dressings can interact with and destroy the bacteria found in exudate [174].

Briefly, recent data provide the following information regarding AgNP skin absorption: (i) there is plenty experimental evidence with respect to the in vitro skin permeation by nanoparticles, and (ii) there is an important increase in permeation in the case of damaged skin [175]. When naturally available biopolymers (e.g., chitosan [176] or collagen [177]) are implied in novel nanotechnology approaches, they possess tremendous potential regarding the obtaining of novel and functionally improved platforms for effective wound-healing applications [178].

Acticoat™ and Bactigras™ (Smith & Nephew), Aquacel™ (ConvaTec), PolyMem Silver™ (Aspen), and Tegaderm™ (3M) are representative biocomposites modified with ionic silver and approved by the United States (US) Food and Drug Administration (FDA) for wound-dressing applications. In addition to these commercial products, promising results were reported with respect to the incorporation of AgNPs within novel and naturally derived biomaterials for enhanced wound-healing management, such as (but not limited to) modified cotton fabrics [179,180], bacterial cellulose [181,182], chitosan [176,183], and sodium alginate [184,185].

The use of AgNPs and Ag^+^ carriers also represents a valuable strategy for delayed diabetic wound-healing processes, since diabetic wounds may be accompanied by numerous secondary infections. AgNPs can help diabetic patients in early wound-healing stages, additionally providing minor scars [186]. Taking into account the efficient and enhanced antibacterial effects exhibited by AgNPs and the impressive interest oriented toward their application in wound therapy and medical-device coatings, their biocompatibility and safety aspects must be thoroughly clarified [187].

## 7. Silver Nanoparticles for Bone Healing

Every year, millions of people worldwide are affected by distinctive and complex bone-related pathologies, including infectious diseases, degenerative and genetic conditions, cancers, and fractures [188]. Unfortunately, the opportunistic contamination and colonization of orthopedic implants represent major concerns in osseous-tissue replacement strategies, since the related infections are associated with high morbidity [189]. Bone is an active tissue that undergoes regenerative and restorative processes through the intrinsic and complex bone-remodeling mechanism [190]. Bone grafts are usually implanted to replace or restore severe defects that irremediably affect osseous tissue, such as genetic malformations, tumors, or traumas [191]. Orthopedic and bone-implant-related infections are usually associated with highly inflammatory processes and subsequent implant loss and bone-destruction phenomena [192].

Previous studies reported that AgNPs naturally improve the differentiation process of MC3T3-1 pre-osteoblast cells and subsequent bone-like tissue mineralization, when compared with other NPs [193]. Currently, silver-coated prostheses represent an unconventional approach during the prophylaxis of tumor-related infections and extensive trauma-related infections. However, no clinical studies comparing the long-term clinical impact of nanosilver-coated implants for revision arthroplasty are reported as of yet [194]. The self-repairing capability of bone can be limited when bacterial activity occurs in bone defects. Compared with usual antibiotics, AgNPs possess intrinsic antibacterial activity with a broader spectrum. Also, the bacterial resistance to AgNP activity is an uncommon phenomenon, thus emphasizing that the bactericidal mechanisms of nanosilver act in synergy. Thanks to this peculiar property, AgNPs have the capability to inhibit or impair biofilm development or mature biofilm, respectively, in the case of antibiotic-resistant bacteria, such as methicillin-resistant *Staphylococcus aureus* [195].

Human bone, dentin, and dental enamel are mainly composed of crystallized hydroxyapatite (HA), which is a calcium-phosphate salt [196]. Given the specific biocompatibility of biosynthesized and synthetic HA, this material and its derivatives are extensively explored for the development of unconventional osseous-related restorative and regenerative strategies, either as artificial bone grafts or as coating materials for metallic implants [197]. With regards to the superficial modification of various metallic implant surfaces, biocompatible HA integrated with silver (either in metallic or ionic form) represents a suitable choice for the fabrication of bioactive and antimicrobial bone implants [198]. The antimicrobial efficiency of HA-based coatings embedded with nanosilver was evidenced against Gram-positive [199,200,201] and Gram-negative [202,203,204] bacterial strains.

In terms of bone-replacement procedures, AgNPs are normally used as doping materials for synthetic and bio-inspired bone scaffolds, with relevant results being recently reported [205,206]. In order to induce antibacterial properties in HA coatings, several experimental techniques proved suitable for the incorporation of nanosilver within calcium-phosphate materials, such as laser-assisted deposition, electrochemical deposition, magnetron sputtering, ion-beam-assisted deposition, sol-gel technology, and microarc oxidation [207].

Previous studies showed that AgNP-implanted titanium displayed improved antibacterial ability, as well as excellent compatibility with osteoblasts, thanks to the micro-galvanic effects produced between the implanted AgNPs and the titanium substrate [208]. Many studies investigated the feasibility and clinical potential of adjusting acrylic cements with AgNPs, in order to provide unconventional and functionally improved biomaterials for orthopedic applications. While previous studies explored different acrylics modified with AgNPs, a significant part of the previous work is limited, since vital material characteristics and mechanical properties were not thoroughly analyzed [159,209,210,211].

Moreover, the beneficial addition of antimicrobial AgNPs within composite matrices designed for bone-tissue engineering were emphasized. In a recent study, it was shown that AgNPs could promote the osteogenesis and proliferation of mesenchymal stem cells (MSCs), in order to enhance the healing process of bone fracture [212]. A correlation was also reported between NP uptake and growth in clathrin-dependent endocytosis in the case of MSCs and osteoblasts, indicating that this route may represent the principal cellular internalization pathway of AgNPs [213]. Taking into account the limited capacity of bone tissue to fully reconstruct or replace severe defects, the development of novel and performance-enhanced implants is required. Thus, new pathways were used to stimulate bone regeneration and also to prevent the side effects correlated with therapeutics currently used in the clinic [214].

## 8. Silver Nanoparticles for Other Medical Applications

Thanks to their unique physiochemical properties and biofunctional features, such as anti-inflammatory, anti-angiogenesis, antiplatelet, antiviral, antifungal, and antibacterial activities, AgNPs play an important role in the development and implementation of novel biomedicinal strategies [45]. Recently, AgNPs were intimately investigated regarding their promising anticancer effects exhibited in different human cancerous cell lines, such as endothelial cells, IMR-90 lung fibroblasts, U251 glioblastoma cells, and MDA-MB-231 breast cancer cells [215,216]. AgNPs possess the intrinsic capability to merge with mammalian cells and to easily penetrate them by means of energy-driven internalization pathways [217]. Another attractive property of AgNPs relies on their specific fluorescence, making them suitable candidates for detection and dose-enhancement purposes in X-ray irradiation applications [218].

At the moment, the combination of therapy and diagnosis, known as theranostics, represents the most important, attractive, and challenging approach embraced by healthcare practitioners and researchers with respect to the effective and personalized therapy of cancer desideratum [219]. AgNPs are also plasmonic structures, capable of particularly scattering and absorbing the light impinging certain areas. After their selective uptake into cancerous cells, AgNP-derived scattered light can be used for imaging purposes, whereas absorbed light can be used for selective hyperthermia [220].

Cardiovascular diseases (CVDs) represent a major cause of worldwide human death, being responsible for more than 17.7 million deaths in 2015 [221]. Recently, many studies focused on the evaluation of the effects of AgNPs on various types of cell encountered in the complex vascular system, but the reported results were contradictory. However, the collected data can provide substantial knowledge with respect to the potential benefits of AgNPs for pathological and physiological stages related to the cardiovascular system, thus contributing to the development of novel and specific molecular therapies in vascular tone, vasopermeability, and angiogenesis [222]. Cardiovascular pathologies, such as hypertension, may influence the toxic effects induced by AgNPs [223]. The first silver-modified cardiovascular medical device was a prosthetic silicone heart valve coated with elemental silver, which was developed to avoid valve-related bacterial infection and to reduce inflammation response [224].

Malaria, one of the most common infectious diseases encountered in tropical and sub-tropical regions, became a major healthcare concern all around the world. It was shown that AgNPs possess powerful activity against both the malarial parasite (*Plasmodium falciparum*) and its related vector (*Anopheles* female mosquito). The intrinsic anti-plasmodial effects exhibited by nanosilver-based compounds and materials represent a solid starting point toward the nanotechnology-derived therapy and worldwide control of malaria [24,225,226].

The human eye is a complex organ, with impressive vascularization and innervation, that can be easily exposed to microbial contamination under proper temperature and humidity conditions [227,228]. Nanosilver-based compounds and materials proved promising potential toward the development of unconventional and performance-enhanced therapy of eye-related infectious conditions. AgNPs coated with calcium indicators proved to have reduced damage with respect to retinal cells, and could be experimentally applied for retinal imaging in a mouse animal model [229,230]. The bactericidal effects related to AgNP-containing nanomaterials are essential aspects which must be further considered for their exploitation as an improved class of antibacterial agent for ocular applications [14,231,232].

AgNPs can be successfully used as novel nanostructured platforms for diagnostics and the treatment of different cancers [233]. The broad-spectrum bioactivity of AgNPs makes them promising agents not only for anti-infective fighting strategies, but also in critical tumor and multi-drug resistance tackling approaches.

## 9. Toxicity of Silver Nanoparticles

Even if AgNPs possess tremendous advantages that recommend them for novel and challenging biomedical applications, their toxicity became an intensive study subject only recently. The daily amount of silver derived from natural sources in food and water ingested by humans is approximately 0.4–30 µg [234]. The available studies performed with respect to the toxic effects exhibited by AgNPs within biological systems, such as bacteria and viruses or human cells, report contradictory and various results [235,236]. AgNPs are generally presented as highly effective antimicrobial agents with non-toxic effects to healthy mammalian cells [237]. However, various in vitro studies demonstrated the nanosilver-related toxic effects in rat hepatocytes and neuronal cells [238], murine stem cells, and human lung epithelial cells [239]. The toxicity of AgNPs was also investigated during in vivo assays. The toxicity studies performed in a rat ear model proved that AgNP exposure resulted in significant mitochondrial dysfunction and subsequent temporary or permanent hearing loss, depending on the inoculation dose. Even low concentrations of AgNPs were absorbed by retinal cells and resulted in important structural disruption, due to the increased number of cells that underwent oxidative stress [240].

**Figure 2:**
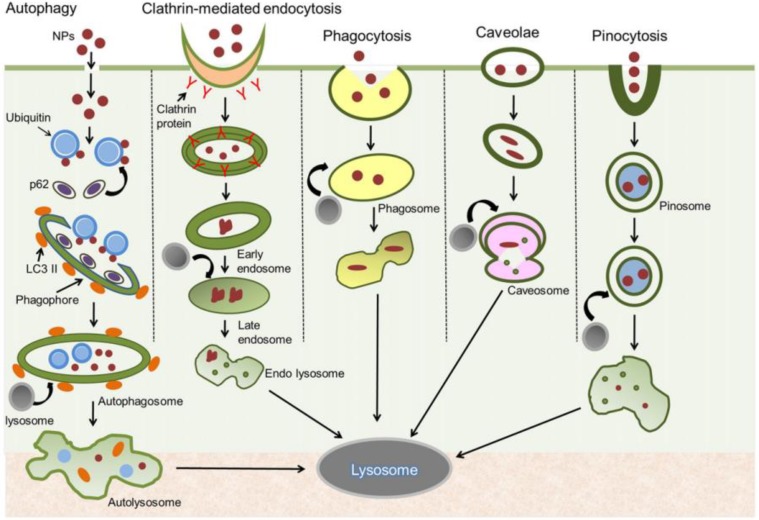
Schematic representation of plausible methods of cellular uptake of AgNPs [243]. Reprinted with permission from [243]. Elsevier, 2015.

The possible toxicity mechanisms related to AgNPs are depicted in Figure 2 [241]. The performed studies also proved that variations in surface charge resulting from the surface functionalization of AgNPs can impact cellular uptake, translocation to various tissues, and cytotoxicity. The magnitude of the surface charge, as measured by the zeta potential, can influence the amount of nanoparticles and their mechanism of uptake into cells [242].

In order to investigate the toxic effects caused by exposure to nanosilver-based systems, thorough assays are required, considering both cellular and animal models. Regarding the in vivo biocompatibility and biodistribution assays, the reported data evidenced that AgNPs can result in structural and physiological alteration of vital organs. For example, inhaled AgNPs may form deposits in the alveolar regions, leading to lung injuries, and may also generate significant modifications within the nervous system, and liver and kidney tissues. Intratracheal instillation of AgNPs can affect vascular reactivity and can further exacerbate cardiac reperfusion/ischemia injury [244,245].

The toxicity of AgNPs is related to their transformation under biological conditions and environmental media, including their interactions with biological macromolecules, surface oxidation, and the release of silver ions. Also, it is very important to precisely distinguish the toxicity rate related to either nanosized silver or ionic silver [246]. Many studies proved that AgNP exposure can induce a decrease in cell viability through different cellular mechanisms. One of these mechanisms is represented by the induction of apoptosis-related genes and the activation of apoptosis mechanism. Also, it was proven that nanosilver can cause the formation and intracellular accumulation of ROS, modification of mitochondrial membrane permeability, and DNA damage [247,248,249]. The in vitro toxicity of AgNPs was investigated in several research studies, but there is still a lack of consistent and reliable data. This is a general concern in nanotoxicology, and more research coherence is needed to produce meaningful results. According to recent data, the main in vitro outcomes occurring upon exposure to AgNPs were reported as increases in oxidative stress, genotoxicity, and apoptosis levels [250,251,252]. AgNPs may induce significant oxidative damage with respect to the cellular membrane and organelles such as the nucleus, mitochondria, and lysosomes, thus leading directly to necrotic or apoptotic phenomena. The oxidative stress caused by AgNPs can result in inflammatory responses, including the activation of innate immunity and increasing the permeability of endothelial cells [253]. AgNPs, inoculated at non-cytotoxic doses, may cause chromosomal abnormality, DNA damage, and possible mutagenicity [254,255,256].

The genotoxicity and cytotoxicity of AgNPs are influenced by several physicochemical features, including dispersion rate, concentration, surface charge, size, morphology, and surface functionalization [257,258]. The physicochemical aspects of nanosilver-based systems and materials mainly distribute and categorize numerous toxicological concerns, and also establish a ladder of toxicity framework while imposing on the biological system. The experimental results reported until recently are insufficient regarding the accurate toxic effects of AgNPs and their related toxicity mechanisms [35,259].

## 10. Conclusions

Silver nanoparticles (AgNPs) are intensively explored nanostructures for unconventional and enhanced biomedical applications, thanks to their size-related attractive physicochemical properties and biological functionality, including their high antimicrobial efficiency and non-toxic nature. AgNP-based nanosystems and nanomaterials are suitable alternatives for drug delivery, wound dressing, tissue scaffold, and protective coating applications. Various physicochemical parameters were related to the intrinsic antimicrobial effects exhibited by AgNPs, such as size, shape, concentration, surface charge, and colloidal state. Moreover, the impressive available surface of nanosilver allows the coordination of many ligands, thus enabling tremendous possibilities with respect to the surface functionalization of AgNPs.

There is a significant amount of research data proving the beneficial effects of AgNPs in novel biocompatible and nanostructured materials and devices developed for modern therapeutic strategies. In addition to their attractive and versatile antimicrobial potential, AgNPs provide additional mechanical, optical, chemical, and biological peculiarities that recommend them for the design, obtaining, evaluation, and clinical assessment of performance-enhanced biomaterials and medical devices. Still, thorough investigations regarding their short-term and long-term toxicity, as well as the responsible toxic-related mechanisms, are required.

The current limitations related to conventional healthcare practice and the latest challenges resulting from nanosilver-based technology outline the impressive potential of silver nanoparticles in biomedicinal applications. Whether we consider the modification of available biomaterials and devices or the development of novel nanostructured ones, AgNPs are ideal candidates for achieving the very close modern biomedicine desideratum.
